# Overestimating Fish Counts by Non-Instantaneous Visual Censuses: Consequences for Population and Community Descriptions

**DOI:** 10.1371/journal.pone.0011722

**Published:** 2010-07-22

**Authors:** Christine Ward-Paige, Joanna Mills Flemming, Heike K. Lotze

**Affiliations:** 1 Department of Biology, Dalhousie University, Halifax, Nova Scotia, Canada; 2 Department of Mathematics and Statistics, Dalhousie University, Halifax, Nova Scotia, Canada; University of Pretoria, South Africa

## Abstract

**Background:**

Increasingly, underwater visual censuses (UVC) are used to assess fish populations. Several studies have demonstrated the effectiveness of protected areas for increasing fish abundance or provided insight into the natural abundance and structure of reef fish communities in remote areas. Recently, high apex predator densities (>100,000 individuals·km^−2^) and biomasses (>4 tonnes·ha^−1^) have been reported for some remote islands suggesting the occurrence of inverted trophic biomass pyramids. However, few studies have critically evaluated the methods used for sampling conspicuous and highly mobile fish such as sharks. Ideally, UVC are done instantaneously, however, researchers often count animals that enter the survey area after the survey has started, thus performing non-instantaneous UVC.

**Methodology/Principal Findings:**

We developed a simulation model to evaluate counts obtained by divers deploying non-instantaneous belt-transect and stationary-point-count techniques. We assessed how fish speed and survey procedure (visibility, diver speed, survey time and dimensions) affect observed fish counts. Results indicate that the bias caused by fish speed alone is huge, while survey procedures had varying effects. Because the fastest fishes tend to be the largest, the bias would have significant implications on their biomass contribution. Therefore, caution is needed when describing abundance, biomass, and community structure based on non-instantaneous UVC, especially for highly mobile species such as sharks.

**Conclusions/Significance:**

Based on our results, we urge that published literature state explicitly whether instantaneous counts were made and that survey procedures be accounted for when non-instantaneous counts are used. Using published density and biomass values of communities that include sharks we explore the effect of this bias and suggest that further investigation may be needed to determine pristine shark abundances and the existence of inverted biomass pyramids. Because such studies are used to make important management and conservation decisions, incorrect estimates of animal abundance and biomass have serious and significant implications.

## Introduction

Underwater visual censuses (UVC) have been used to enumerate fish *in-situ* in a wide range of areas and habitats since the 1950's. Today, UVC made by scuba divers are standard techniques used to estimate the abundance of animals in the field. Fish counts derived from UVC censuses have been used to describe and monitor spatial and temporal trends in populations and communities that include mobile fishes [1]–[5]. Recently, these techniques have been deployed in remote oceanic atolls to describe the community structure of relatively pristine reefs [6]–[9]. These studies have provided invaluable insight into the effect of exploitation on fish communities; however, the absolute values appear high – even for pristine reefs. For example, densities of ∼100,000–500,000 top predators·km^−2^ (including sharks, jacks and snappers) were reported for the Line Islands [9]. In contrast, the density of epaulette sharks in Australia is 3,000–12,000 individuals·km^−2^ [10], lions in Tanzania is about ∼0.08–0.13 individuals·km^−2^ [11] and what is considered a high density of cattle on grasslands is 83 individuals·km^−2^ [12]. Here, we explore possible reasons for high estimates of marine top predator density and biomass that is related to sampling procedures.

Today, the belt-transect and stationary-point-count techniques are used regularly to estimate the density and biomass of underwater organisms [13]–[16]. In the belt-transect technique, one or two divers swim along a straight line and record the animals they observe within a fixed distance of the line [17]. In the stationary-point-count technique, the diver remains still and records the fish observed within a fixed distance of the diver for a certain amount of time. Commonly, fish counts are converted to density by standardizing by the area sampled. This calculation is suitable for stationary organisms such as corals, plants, and slow-moving invertebrates which are unlikely to leave or enter the sample area during the survey. In these cases, surveys produce reliable density estimates because they are essentially instantaneous counts, and the same result would be obtained if the survey was conducted instantaneously or over longer periods of time. For mobile animals like fish, however, counts are highly dependent on the technique used. Ideally, researchers use instantaneous censuses and do not count animals that enter the survey area after the survey has started. In practice, however, animals fully or partly entering the survey area within the surveyors view are often counted (personal communication with >30 researchers using UVC). In these cases, simple number-per-area calculations may result in inaccurate density estimates and related population and community descriptions.

Through our discussions with researchers it was generally acknowledged that counting fish that enter the survey boundaries after the census started could generate bias in the counts; however, this bias was considered to be insignificant and acceptable as long as the methods were constant between surveys. Studies using UVC usually deploy the same techniques within each study (e.g. belt-transect with constant width and length) to generate data that are directly comparable. Numerous studies have investigated bias, imprecision and variability in counts from visual censuses [18]–[23] and have focused on fish behaviour (e.g. reaction to diver), detection, misidentification, survey effort, site selection and recounting. However, none have addressed the accuracy of counting mobile fish in non-instantaneous UVC.

The importance of fish mobility (e.g. direction) on UVC was previously investigated by Watson and Quinn [24]. Using a simulation program the authors concluded that the speed at which the fish approached the belt-transect diver (not the stationary-point-count diver) caused the most appreciable bias between the observed and true density. However, there were a few differences between the assumptions of their simulation and the practice of the sampling protocols investigated in the current study. Most importantly, for the belt-transect technique the simulated divers did not count fish that entered the survey area within the diver's view. In practice, however, targeted fish that enter the transect area in front of the diver are commonly recorded (personal communications with >30 researchers commonly using UVC). Additionally, for the stationary-point-count technique simulated by Watson and Quinn [24], the diver surveyed the area from above (i.e. looking down) and did not record fish that entered the survey area after the survey started. In practice, however, stationary-point-count divers often remain in the middle of the circle and turn in one direction while conducting a survey [25]. Because the diver deploying the stationary-point-count technique remains still it is thought to be a superior sampling method for censusing mobile fishes because it allows the fish to acclimate to the diver's presence and move back into their original positions – within the survey boundaries [25].

In this study, our aim was to evaluate the bias caused by fish speed in non-instantaneous UVC. Without accounting for this factor, the bias caused by animal detectability and behaviour (e.g. drawn towards the diver) that exists in instantaneous surveys may be compounded by methodological bias and could lead to unrealistic density, biomass, and community descriptions. Here, we developed a model in R [26] to simulate fish and divers deploying the belt-transect and stationary-point-count UVC techniques. Because sharks are likely the most conspicuous and mobile fishes detected during UVC, we tailored the model to simulate fish speeds to those reported for sharks. We investigate the bias between the observed and expected counts across a range of fish speeds in non-instantaneous UVC. Then, we explore the bias produced by different survey procedures (visibility, survey time, transect width, transect diver speed, stationary radius) and fish mobility (speed and turning angles). Finally, using examples from the scientific literature, we demonstrate the effect these biases may have on abundance, biomass and community descriptions.

## Methods

### Model Description

**Figure 1:**
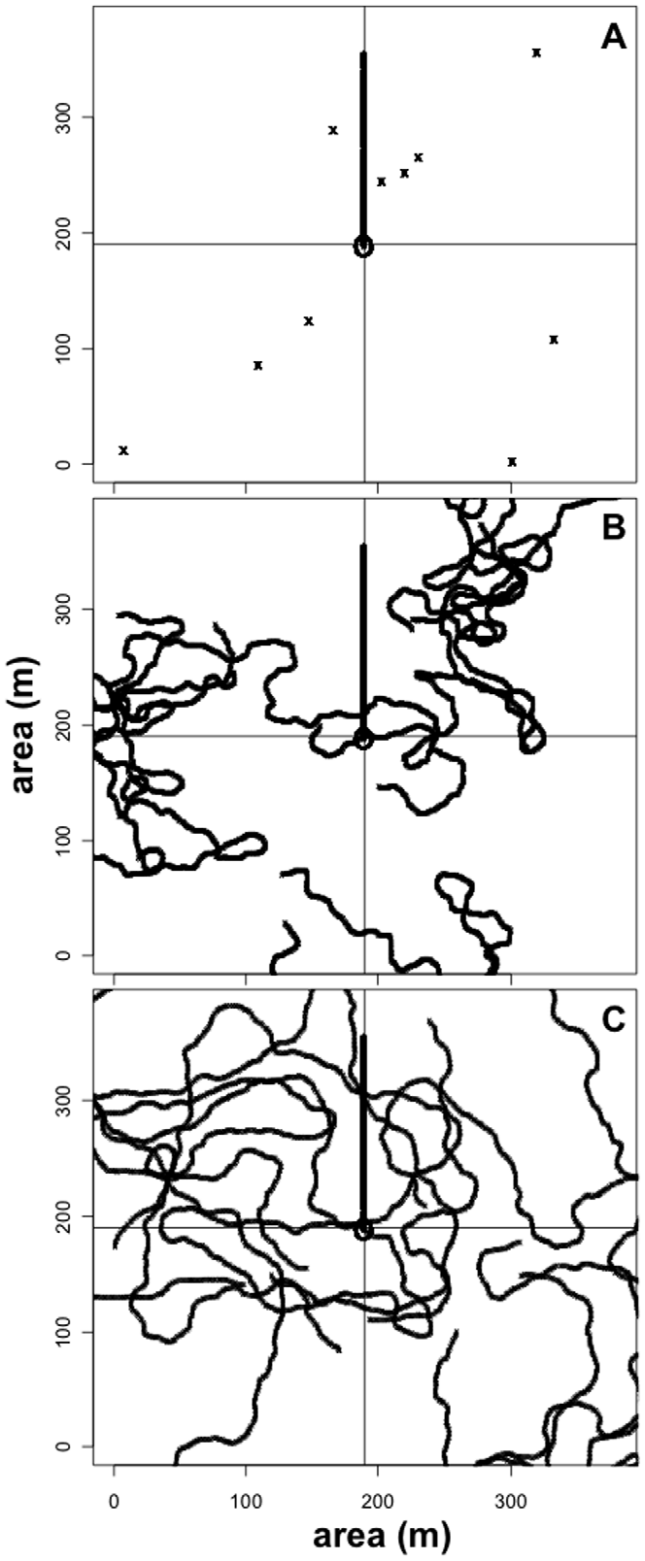
Demonstration of the simulation model *AnimDens* showing the movement of divers and fish. Two divers were simulated, the stationary-point-count diver remained in the centre of the sampling area (circle) and the belt-transect diver followed a straight path (bold solid line). Fish speeds of 0, 0.5 and 1.0 m·s^−1^ (top to bottom) are shown.

**Figure 2:**
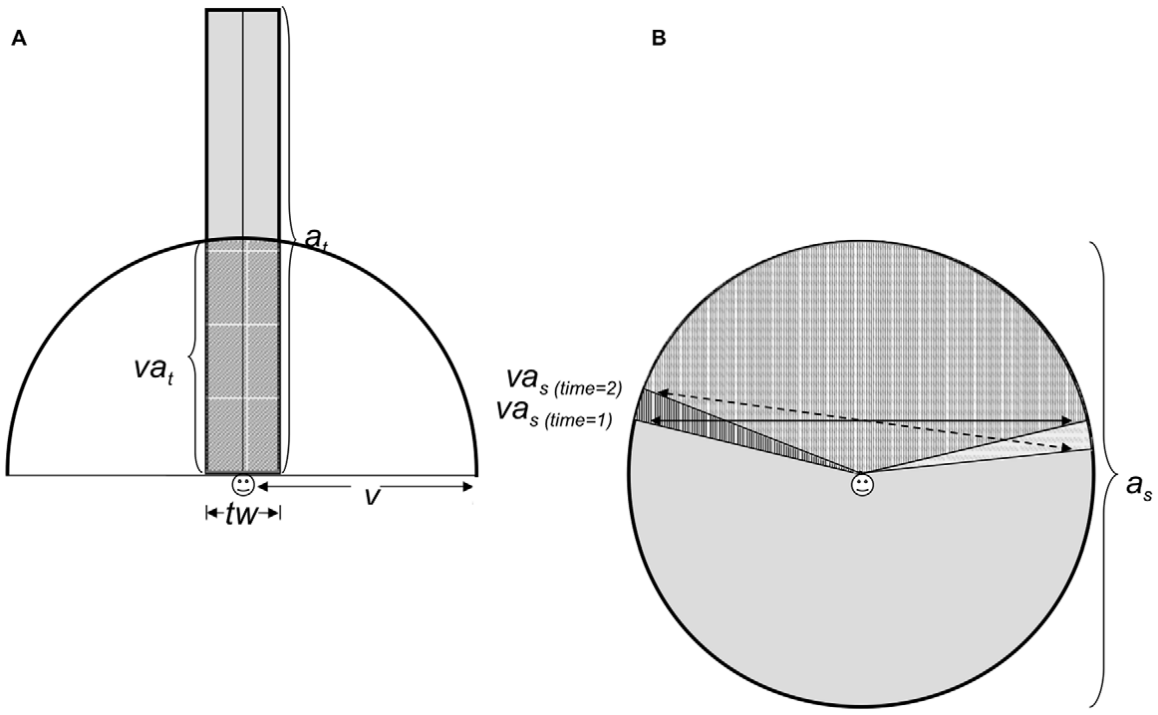
Diagram of the visual sampling field for the belt-transect diver (left) and the stationary-point-count diver (right). Symbols: *a_t_* is the total area surveyed by the belt-transect diver; *va_t_* is the area visually surveyed by the belt-transect diver in one time step; *tw* is the width of the belt-transect; *v* is the visibility distance; *a_s_* is the total area surveyed by the point count diver; *va_s_* is the area visually surveyed by the point count diver in one time step.

Our model *AnimDens* was written in R [26] to simulate divers counting fish while deploying the belt-transect and stationary-point-count UVC techniques (File S1). Two experiments were used to explore the effect of fish mobility and survey procedure on the difference between observed and expected counts. Simulations were run across a range of fish speeds and survey procedures to determine the effect of these parameters on observed counts. Figure 1 shows sample runs for fish that remained still, that moved at 0.5 m·s^−1^, and at 1.0 m·s^−1^. For simplicity, the model assumed a sample area that was featureless, flat and 1 m deep. For each simulation, a diver from each of the two census methods was placed in the centre of the sample area and each had an orientation of 90° (each facing in the same direction) at initial time, t_0_. The sample area was populated with fish with a random distribution and random initial orientation. At t_0_ the number of fish observed and recorded by each diver was a function of the distance and the angle between the diver and each fish (those located within view of the diver). For the belt-transect diver, the distance was set to maximum visibility *v* and an angle of ±90° but only to a distance of transect width (*tw*) to the right and left of the diver's location (Fig. 2A). Because the belt-transect diver sampled an area directly in front of them the angle was set to 180°. For the stationary-point-count diver, all fish within ±80° of the diver's main orientation were counted (Fig. 2B). Fish that reached the area boundaries were allowed to leave and return (i.e. not reflected back into the sample area). Note that the simulated divers did not recount fish they already recorded (as if they were all numbered), as divers strive to do in the field [17], [21].

After each time step, the fish moved with a given speed in a restricted randomly selected direction (turning angle) of their last orientation (correlated random walk). Although fish often travel at variable speeds with complex behaviours, the concern here is the chance of the fish entering the sample area during the census (e.g. 5 minutes) within the diver's field of view. Once the fish enters the sample area it is recorded and its speed and behaviour is no longer relevant and therefore the fish speed and directionality remained constant through each simulation. In reality, divers continuously view the survey area; however, to speed up the simulation process we set the time step to two seconds (i.e. fish were counted every two seconds), which leaves the slight possibility of fish being undetected. For the stationary-point-count we followed the sampling protocol outlined in Bohnsack and Bannerot [25] and set the divers to remain still and to turn slowly in one direction (here set to +4°) in each time step. Although the belt-transect technique usually does not specify the survey time or swim speed of the diver in published methodology [9], [18], [22] we set the belt-transect diver to move forward at a range of speeds, which are reasonable for counting and recording conspicuous fish.

Experiment 1 examined the effect of fish speed alone. Here we fixed all other parameters to commonly reported values and only varied fish speed. Here, fish density was fixed at 0.2 fish·m^−2^, approximately the maximum apex predator density reported by UVC [9]. Fish speeds included 0, 0.001, 0.01, 0.1, 0.2, 0.4, 0.6, 0.8, 1.0, 2.0, 4.0 m·s^−1^, and were based on those attained by sharks. For example, *Ginglymostoma cirratum* Müller and Henle (nurse shark) often rest on the bottom and therefore have swimming speeds of 0 m·s^−1^. Other sharks, including *Negaprion brevirostris* Poey (lemon), *Sphyrna tiburo* Linnaeus (bonnethead), *Carcharhinus amblyrhynchos* Bleeker (grey reef) and *Carcharhinus melanopterus* Quoy and Gaimard (blacktip reef) sustain swimming speeds of 0.77–1.29 m·s^−1^ [27], [28]. *Carcharhinus leucas* Müller and Henle (bull) has a burst swimming speed of up to 5.3 m·s^−1^ [29]. Fish turning angles (restriction of the amount that fish were able to turn between time steps) were set to 45°, based on C. Ward-Paige's personal observations of reef sharks (*Carcharhinus perezi* Poey, *Carcharhinus limbatus* Müller and Henle, *C. melanopterus*). Few studies report visibility distance in their published survey methods, although it is expected that surveys would not be conducted under conditions of limited visibility (less than the width of the belt-transect or the radius of the stationary-point-count). Here, we set visibility distance to 13 m, which is the average visibility reported by divers to Reef Environmental Education Foundation (REEF: www.reef.org). Transect width was set to 4 m, which is a commonly used transect width for mobile fishes [7]–[9] and the stationary-point-count distance was set to 7.5 m, a commonly used radius [25]. Although, most published methods do not specify the survey time or swim speed of the belt-transect diver [8], [9], [18], [22], we set survey time to 300 s which is a commonly reported deployment time used for the stationary-point-count and belt-transect methods [7], [19], [21] and diver speed to 4 m·min^−1^, which is a reasonable speed for counting conspicuous fishes. This was run as individual models 30 times each (i.e. 30 replications).

Experiment 2 examined the overall patterns of bias produced by fish mobility (speed and turning angle) and survey procedure (i.e. visibility distance, survey dimensions, diver speed, and survey time) on observed counts *AnimDens* was also run across a range of all variables (Table 1). Because of the extent of computing time, we ran 1 simulation for each variable combination. Here, fish speeds were set to the same values as before. Fish density was fixed at 0.1 fish·m^−2^. Fish turning angles ranged from 1° (direct walk: turning very little) to 45° (turning a lot) which is similar to that described by Papastamatiou et al. [30]. Survey times and transect widths cover a wide range of values. Visibility distances cover a range of values reported in the Reef Environmental Education Foundation (REEF; www.reef.org) database.

**Table 1:** Levels of each predictor variable used to examine the bias in fish counts produced by fish speeds and survey procedures.

Fish speed (m·s^−1^)	Survey time (s)	Visibility (m)	Transect width (m)	Diver speed (m·min^−1^)	Fish turning angle (°)
0	60	10	1	1	1
0.001	300	20	2	4	22.5
0.01	600	30	4	7	45
0.1	900	40	5		
0.2	1200		8		
0.4	1800		10		
0.6			20		
0.8					
1					
2					
4					

Each combination of the values was run for 1 simulation.

### Analyses

The results (i.e. counts made by each diver) of each model simulation was used to compare observed count (*c_s,t_*) and expected count, which were then used to examine trends in bias through the range of survey procedures and fish speeds.

Expected count (*x_s,t_*) was calculated as:
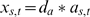
where (*d_a_*) is the true density (number of fish divided by the total area entered in the simulation) and *a_s,t_* is the area surveyed by the stationary-point-count (*a_s_*) or belt-transect (*a_t_*) divers. The area surveyed was calculated for each UVC method as:
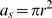


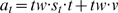
where *r* is the sampling distance (radius) used in the stationary-point-count technique, *tw* is the transect width, *s_t_* is the swimming speed of the belt-transect diver, *t* is the survey time and *v* is the visibility distance (see Fig. 2 for a visual description of the survey variables). The length of the swim path for the belt-transect diver was a function of swimming speed (*s_t_*) and survey time (*t*).

The relative bias (*b_s,t_*) for each UVC method was computed as:
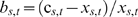
The mean relative bias for each model was used to examine how fish speed and survey procedures bias observed densities.

## Results

**Figure 3:**
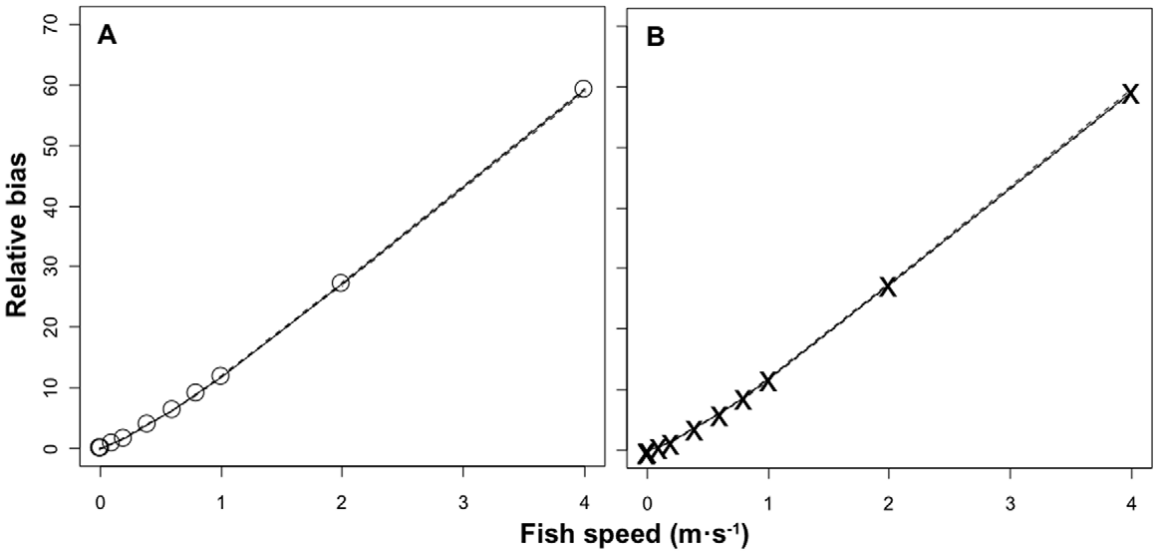
Comparison of fish speed with relative bias [(observed count – expected count)/expected count] for belt-transect (A) and stationary-point-count (B) divers. Simulation conditions were set as: survey time = 300 s, visibility = 13 m, transect width = 4 m, stationary-point-count radius = 7.5 m, diver speed = 4 m·min^−1^, and fishing turning angle = 45°. Shown are the mean values across 30 simulations (x and o) with standard errors (dashed lines).

Experiment 1 shows that relative bias for both the belt-transect and stationary-point-count divers increased with fish speed and followed the same pattern and range for both survey techniques under the specified sampling conditions (Fig. 3). Even counts of very slow moving fish (e.g. 0.01 m·s^−1^) were overestimated. For the belt-transect survey the relative bias increased from −0.04 to 11.84 for fish moving at 0.001 and 1.0 m·s^−1^, respectively. For the stationary-point-count diver, bias increased from −0.0004 to 11.89 for fish moving at 0.001 and 1.0 m·s^−1^, respectively. Thus, over a 300 s survey time, fish moving at 1.0 m·s^−1^, a typical speed for reef sharks, were overestimated by more than an order of magnitude (Fig. 3) by both UVC techniques. For faster moving fish (4 m·s^−1^), the bias increased up to 60 (Fig. 3). The standard errors (Fig. 3) show that there is little variation between simulations.

**Figure 4:**
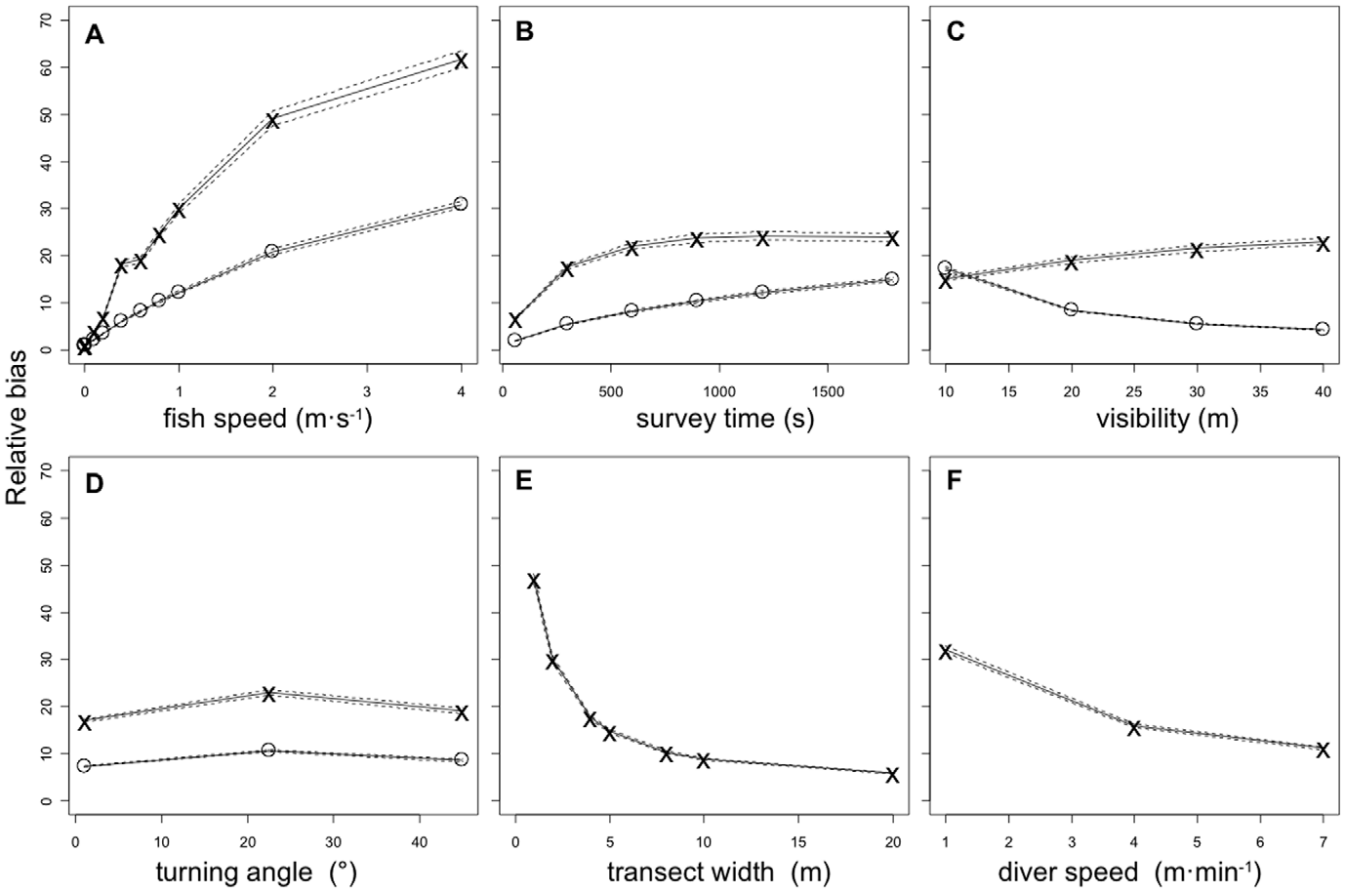
The effect of each predictor variable on the average relative bias (solid lines), dashed lines represent ± S.E, across all model combinations. See Table 1 for details on predictor variables. x = Belt-transect diver, ⋄ = Stationary-point-count diver. Panels E and F are only applicable to the belt-transect.

Across all model combinations, fish speed, fish turning angle and survey procedure had varying effects on bias (Fig. 4). Overall, fish speed had the greatest effect on bias that increased systematically with fish speed up to 61 for the belt-transect and 31 for the stationary-point-count diver for fish moving at 4 m·s^−1^. Survey time and visibility affected the two survey techniques differently. With longer survey times, the belt-transect diver covers more area while the stationary-point-count diver does not. Bias generally increased with survey time. Under scenarios of increased visibility, the belt-transect diver surveys more of the transect at a given moment but does not increase the area covered (except at the end of the survey). For the stationary-point-count technique, visibility distance represents the radius that is being sampled and therefore increases the area covered. Therefore, bias increased with visibility distance for the belt-transect diver and decreased for the stationary-point-count diver. There was minimal effect of fish turning angle on the overall bias indicating that the effect of directionality (whether they turn a lot or a little – *not* if they are drawn towards or pushed away from the diver) is marginal. The area covered by the belt-transect diver increases with transect width and diver speed. Therefore, as both factors increased the overall bias was reduced. The biases for each combination of the survey parameters (Table 1), 16,632 models in total, are listed in Table S1.

## Discussion

Our results indicate that mobile fish are over-counted in non-instantaneous UVC as an effect of them entering the survey area after the survey starts, not recounting. In general, relative bias increased with fish speed but the extent depended on the UVC technique and survey procedure used. Therefore, setting aside all issues of detection, misidentification, recounting and behaviour of the animal in response to the diver (whether they are drawn toward or repelled away from the diver), counts obtained by non-instantaneous UVC only provide approximate estimates of true values (e.g. density) for stationary and very slow moving (<0.001 m·s^−1^) animals. For mobile animals, however, caution needs to be applied when using non-instantaneous UVC and the implication of the bias depends on the goal of the study.

When the goal of a scientific study is to examine relative spatial and temporal differences in the density of a single species surveyed under homogenous sampling conditions, non-instantaneous UVC count data may produce satisfactory information. For example, Robbins et al. [14] utilized constant belt-transect surveys to compare relative abundance of *Triaenodon obesus* Rüppell (whitetip reef) and *Carcharhinus amblyrhynchos* (grey reef) sharks along a gradient of fishing pressure. Their conclusions should be valid regardless of the surveys being conducted instantaneously or not, as long as the fish had similar levels of mobility between sites (e.g. not resting and feeding grounds), surveys were conducted by consistent UVC methods, and assuming that the fish had the same detectability and behaviour towards divers in all locations. However, the technique used (i.e. instantaneous or non-instantaneous) would affect the values of absolute density and all descriptions that are based on these values (e.g. biomass and community structure).

**Figure 5:**
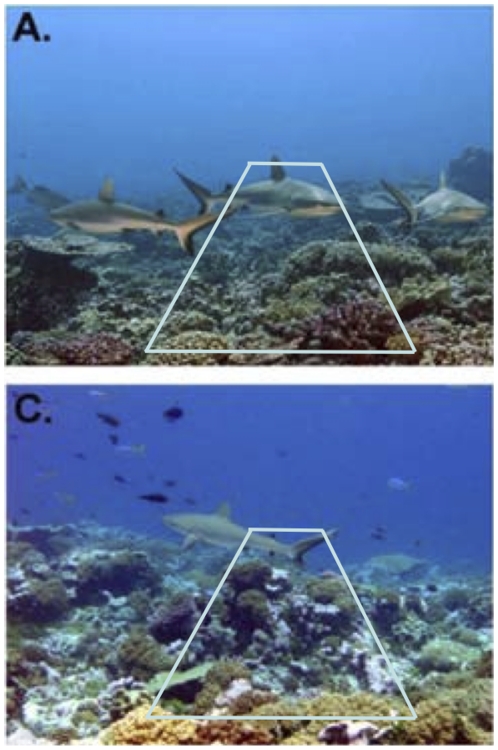
Illustration of an instantaneous count (snapshot) of sharks in a belt-transect UVC (photos from **Fig 2A and 2C** in Sandin et al. 2008). To visualize the area that would be covered by a belt-transect, we have drawn in the approximate survey boundaries of a 50 m^2^ transect (∼4 m wide×12.5 m long which is represented by visibility distance). Sandin et al [9] covered 100 m^2^ (4 m wide×25 m long) per transect – approximately double the area outlined.

Patterns in absolute density are often extrapolated from observed fish counts obtained by UVC, yet whether or not UVC were done instantaneously is rarely reported. We illustrate this point using the data and photos shown of Kingman and Palmyra atolls – the two locations where sharks dominated the top predator biomass and where the highest top predator density and biomass has been reported for reefs [9]. Photos are essentially ‘instantaneous snapshots’ of the reef and represent counts made by instantaneous UVC techniques (Fig. 5). In both photos, one top predator (i.e. shark) occurred within ∼50 m^2^ – a density of 0.02 individuals·m^−2^. However, Sandin et al. (2008) reported densities of ∼0.2 individuals·m^−2^ for both Kingman and Palmyra, which corresponds to 10 individuals per 50 m^2^ belt-transect. If we assume that the most top-predator-rich photos were used to demonstrate their abundance on reefs at Kingman and Palmyra, then the density would have been overestimated by one order of magnitude. Although a number of factors may have contributed to the discrepancy between the data and figures shown in Sandin et al. [9] (e.g. site selection, fish behaviour, schooling), our results suggest that the difference could be explained by fish speed alone if non-instantaneous surveys were used.

In most cases where non-instantaneous UVC have been conducted, the true instantaneous density is unknown. In these cases, the biases given in Table S1 may be used to explore the effect that fish speed could have on observed densities under different survey procedures. For example, Sandin et al. [9] used a belt-transect width of 4 m and covered 100 m^2^. Table 2 shows the section of Table S1 that corresponds to these values for a range of fish speeds, with assumptions of visibility = 20 m (presumably a conservative value for the Line Islands), survey time = 300 s, fish turning angle = 45°, diver swim speed = 1 m·min^−1^. Based on the photographs shown in Sandin et al. [9] the sharks appear to be grey reef sharks (*C. amblyrhynchos*). Although the swimming speed of *C. amblyrhynchos* likely varies, we presume that based on their large-scale movements [28] and the swimming speed of other mobile reef sharks like *Carcharhinus leucas* Müller and Henle (bull), *Carcharhinus melanopterus* Quoy and Gaimard (blacktip reef) and *Negaprion brevirostris* Poey (lemon) a reasonable swimming speed would be ∼1 m·s^−1^. Therefore, if top predators at Kingman included *C. amblyrhynchos*, the bias would be 20.5 (Table 2). Thus, a better estimate of true density may be obtained by standardizing the observed value by the bias plus one (to get the factor of the bias), which gives a density of 0.009 individuals·m^−2^ – 9,000 individuals·km^−2^ which is 191,000 fewer individuals·km^−2^ than the density reported. This density estimate is still high compared to densities reported for no-entry zones on the Great Barrier Reef, which puts grey reef sharks at a density of <250 individuals•km^−2^ [14], however, it may be reasonable since the top predator group in Kingman also includes snapper and jack.

**Table 2:** Relative bias for different fish speeds for belt-transect surveys where survey time = 300 s, transect width = 4 m, visibility distance = 20 m, diver speed = 1 m·min^−1^, fish turning angle = 45°.

Fish speed (m·s^−1^)	Relative bias
0	0.01
0.001	0.03
0.01	0.13
0.1	1.28
0.2	2.92
0.4	6.72
0.6	10.99
0.8	15.47
1	20.51
2	47.99
4	107.97

The diver visually surveyed an area of 100 m^2^.

Because bias increases with fish speed additional problems occur when animals of different mobility are compared to each other. For example, on Kingman atoll, densities of top predators (sharks, jacks and snappers) and all other fishes combined (Carnivores, Planktivores, Herbivores) were reported as ∼0.2 and ∼3.7 fish·m^−2^, respectively [9]. Because various species have different swim speeds, their densities may be overestimated to different degrees. Thus, their relative contribution to the overall community structure becomes skewed. Moreover, densities are regularly converted into standing stock biomass to compare the biomass of trophic levels and sites that have different fish assemblages [3], [6]–[8], [31]. Since the most mobile fishes tend to be the largest, the effect of bias caused by fish speed would be magnified when comparing biomasses of different trophic levels. For example, UVC data revealed the presence of predator dominated ecosystems, or inverted trophic biomass pyramids (higher top predator biomass compared to other trophic levels), in a few relatively unexploited reefs [6]–[9], [31] – a phenomenon that has been rarely demonstrated in other ecosystems [32]–[34]. At Kingman atoll, 85% of the total fish biomass was reported to be top predators – ∼4 t·ha^−1^ for top predators compared to ∼0.8 t·ha^−1^ for other fish [9]. Disregarding the fact that part of the shark is outside of transect boundaries in an instantaneous snapshot (Fig. 5), and using the same sampling conditions as described above and biases from Table 2 the biomass of top predators moving at ∼1 m·s^−1^ may have been closer to 0.187 t·ha^−1^. On the other hand, the ‘other’ fish category comprises many different fish groups (Carnivores, Planktivores, Herbivores) that travel at variable speeds. Unfortunately, there is very limited information on the swimming speed of most reef fishes. A few reef fish species (e.g. damselfish, anemonefish) have reported field swimming speeds of up to 0.2 m·s^−2^ [35]; however, these species have such small home ranges that their effective swimming speed is approximately zero for the purposes of this study (unlikely to enter or leave the survey). Although many of the ‘other’ sampled fish may have swimming speeds faster than 0.2 m·s^−2^, it is possible that the average swimming speed of the ‘other’ fish combined would have been ∼0.2 m·s^−2^ or slower. If this was the case then the ‘other’ fish biomass may have been ≥0.204 t·ha^−1^, more than that reported for the top predator fish category – a bottom-heavy trophic biomass pyramid.

Although the effect of overestimating biomass would be greatest for the largest and most mobile fishes, like sharks, it would also occur for smaller and less mobile fishes such as parrotfish and grouper. For example, Mumby et al. [5] compared predator (mostly groupers) and parrotfish biomass within and outside of the Exuma Cayes Land and Sea Park, Bahamas. Their results showed that *Epinephelus striatus* (Nassau grouper) and parrotfish biomass were higher within the park than outside the park. This statement is likely accurate, assuming the sampling conditions were constant between sites (including visibility and diver speed) and that these fishes maintained the same level of mobility between the sites. However, because grouper and parrotfish have different levels of mobility, if non-instantaneous surveys were used then comparison of their biomass may not be made without accounting for mobility and survey effort.

Our results may also extend to studies that have surveyed communities to obtain species richness – where comparisons are made of animals with different levels of mobility. For example, UVC have been used to compare species richness among sites [36]–[38]. If non-instantaneous surveys were used to compare sites that had different proportions of sedentary and mobile animals (e.g. groupers versus snappers or damselfish versus surgeonfish) then, compounded with the differences in behaviour and detection, bias attributed to mobility would be disproportionate and would lead to inaccurate comparisons due to the methods alone. This may explain why Tittensor et al. [36] and Sandin et al. [9] have contrasting results in fish diversity across a gradient in human disturbance in the Line Islands [9]. The same effect would apply to studies that compared densities of fish in different life stages that have different levels of mobility.

Although not stated explicitly in the scientific literature, based on our inquiries, non-instantaneous visual surveys are used widely. Our results show that non-instantaneous census data do not produce reliable estimates of true density and therefore they should only be used to compare relative differences within species. Variation in survey technique from instantaneous to non-instantaneous, or vice versa, through time or space, may have overstated or dampened observed changes. However, since these surveys provide valuable baseline and monitoring data that have been collected for years, if not decades, it may be advantageous to continue to collect data in the same manner to ensure they remain comparable. As well, non-instantaneous surveys are beneficial for rare mobile species, like sharks, because they increase their chance of detection. However, for absolute values (i.e. density or biomass) given the huge bias that is produced for mobile fish, other survey techniques such as mark-recapture (i.e. photo ID or artificial marks), which are currently used for whale sharks [39]–[41] as well as white [42], [43], sicklefin lemon [44], and grey nurse sharks [45], [46] may produce more accurate estimates of absolute density for highly mobile and rare animals.

The current application of our simulation model was kept simple, but additional complexities of fish behaviour, habitat, sample area, and survey conditions could be included in *AnimDens*. For example, in areas where sharks are thought to be attracted to divers an interaction between the fish and the diver might be more appropriate than a correlated random walk. *AnimDens* may also be useful for investigating other sampling techniques, such as other diver techniques, visual surveys, video censuses, or possibly other sampling procedures entirely (e.g. exploring the likelihood of capturing an animal in a mark-recapture study). The simple version of *AnimDens*, as it is described here, may be useful for obtaining rough estimates of bias and for making order of magnitude adjustments in observed counts, densities, and biomass estimates. However, further complexities should be used when the information is available.

Overall, our simulation study indicates that the difference between instantaneous and non-instantaneous counts of mobile fish can be significant. Therefore, we urge that the treatment of mobile fish during a census must be reported in the scientific literature. Moreover, if non-instantaneous UVC have been used, survey procedures need to be accounted for when estimating density or biomass of mobile species. Studies that have reported results based on non-instantaneous surveys may need to be reanalyzed to determine if the general conclusions remain. In cases where original observations are absent (e.g. video transects), our simulation model *AnimDens* may be used to evaluate possible biases for species of different mobility under different survey procedures. Overall, our results have significant consequences for management and conservation decisions because they demonstrate that in some cases densities of highly mobile species, such as sharks, may be much less than reported. However, accurate estimates of fish density and biomass are essential to set reasonable management and conservation targets. Overestimates can lead to enlarged quotas for exploitation as well as inadequate protection status.

## Supporting Information

File S1 Code for the simulation AnimDens (written in R).
(0.01 MB TXT)

Table S1 Bias results produced by AnimDens
(2.44 MB XLS)

## References

[1] Paddack MJ, Reynolds JD, Aguilar C, Appeldoorn RS, Beets J (2009). Recent Region-wide Declines in Caribbean Reef Fish Abundance.. Current Biology.

[2] Forrester GE, Steele MA, Samhouri JF, Evans B, Vance RR (2008). Spatial Density Dependence Scales up but Does Not Produce Temporal Density Dependence in a Reef Fish.. Ecology.

[3] Harborne AR, Mumby PJ, Kappel CV, Dahlgren CP, Micheli F (2008). Tropical coastal habitats as surrogates of fish community structure, grazing, and fisheries value.. Ecological Applications.

[4] McClanahan TR, Graham NAJ, Calnan JM, MacNeil MA (2007). Toward pristine biomass: Reef fish recovery in coral reef marine protected areas in Kenya.. Ecological Applications.

[5] Mumby PJ, Dahlgren CP, Harborne AR, Kappel CV, Micheli F (2006). Fishing, trophic cascades, and the process of grazing on coral reefs.. Science.

[6] DeMartini EE, Friedlander AM, Sandin SA, Sala E (2008). Differences in fish-assemblage structure between fished and unfished atolls in the northern Line Islands, central Pacific.. Marine Ecology-Progress Series.

[7] Friedlander AM, DeMartini EE (2002). Contrasts in density, size, and biomass of reef fishes between the northwestern and the main Hawaiian Islands: the effects of fishing down apex predators.. Marine Ecology-Progress Series.

[8] Stevenson C, Katz LS, Micheli F, Block B, Heiman KW (2007). High apex predator biomass on remote Pacific islands.. Coral Reefs.

[9] Sandin SA, Smith JE, DeMartini EE, Dinsdale EA, Donner SD (2008). Baselines and degradation of coral reefs in the northern Line Islands.. Plos One.

[10] Heupel MR, Bennett MB (2007). Estimating abundance of reef-dwelling sharks: A case study of the epaulette shark, Hemiscyllium ocellatum (Elasmobranchii : Hemiscyllidae).. Pacific Science.

[11] Creel S, Creel NM (1997). Lion density and population structure in the Selous Game Reserve: Evaluation of hunting quotas and offtake.. African Journal of Ecology.

[12] Gutman M, Holzer Z, Seligman NG, Noy-Meir I (1990). Stocking density and production of a supplemental beef herd grazing yearlong on Mediterranean grassland.. Journal of Range Management.

[13] Hawkins JP, Roberts CM (2004). Effects of artisanal fishing on Caribbean coral reefs.. Conservation Biology.

[14] Robbins WD, Hisano M, Connolly SR, Choat JH (2006). Ongoing collapse of coral-reef shark populations.. Current Biology.

[15] Eggleston DB, Dahlgren CP, Johnson EG (2004). Fish density, diversity, and size-structure within multiple back reef habitats of Key West National Wildlife Refuge.. Bulletin of Marine Science.

[16] Dulvy NK, Freckleton RP, Polunin NVC (2004). Coral reef cascades and the indirect effects of predator removal by exploitation.. Ecology Letters.

[17] Brock VE (1954). A preliminary report on a method of estimating reef fish populations.. Journal of Wildlife Management.

[18] Sale PF, Sharp BJ (1983). Correction for bias in visual transect censuses of coral reef fishes.. Coral Reefs.

[19] Cheal AJ, Thompson AA (1997). Comparing visual counts of coral reef fish: implications of transect width and species selection.. Marine Ecology-Progress Series.

[20] Watson RA, Carlos GM, Samoilys MA (1995). Bias Introduced by the Nonrandom Movement of Fish in Visual Transect Surveys.. Ecological Modelling.

[21] Thresher RE, Gunn JS (1986). Comparative analysis of visual census techniques for highly mobile, reef-associated piscivores (Carangidae).. Environmental Biology of Fishes.

[22] Samoilys MA, Carlos G (2000). Determining methods of underwater visual census for estimating the abundance of coral reef fishes.. Environmental Biology of Fishes.

[23] Lincoln-Smith MP (1989). Improving Multispecies Rocky Reef Fish Censuses by Counting Different Groups of Species Using Different Procedures.. Environmental Biology of Fishes.

[24] Watson RA, Quinn TJ (1997). Performance of transect and point count underwater visual census methods.. Ecological Modelling.

[25] Bohnsack JA, Bannerot SP (1986). A stationary visual census technique for quantitatively assessing community structure of coral reef fishes..

[26] R CDT (2005). R: A language and environment for statistical computing..

[27] Webb PW, Keyes RS (1982). Swimming kinematics of sharks.. Fisheries Bulletin.

[28] Heupel MR, Simpfendorfer CA, Fitzpatrick R Large-Scale Movement and Reef Fidelity of Grey Reef Sharks.. Plos One.

[29] Gray J, Moore JR (1971). How fishes swim;.

[30] Papastamatiou YP, Lowe CG, Caselle JE, Friedlander AM (2009). Scale-dependent effects of habitat on movements and path structure of reef sharks at a predator-dominated atoll.. Ecology.

[31] Newman MJH, Paredes GA, Sala E, Jackson JBC (2006). Structure of Caribbean coral reef communities across a large gradient of fish biomass.. Ecology Letters.

[32] Gasol JM, del Giorgio PA, Duarte CM (1997). Biomass distribution in marine planktonic communities.. Limnology and Oceanography.

[33] Buck KR, Chavez FP, Campbell L (1996). Basin-wide distributions of living carbon components and the inverted trophic pyramid of the central gyre of the North Atlantic Ocean, summer 1993.. Aquatic Microbial Ecology.

[34] Piontkovski SA, Williams R, Melnik TA (1995). Spatial Heterogeneity, Biomass and Size Structure of Plankton of the Indian-Ocean - Some General Trends.. Marine Ecology-Progress Series.

[35] Johansen JL, Fulton CJ, Bellwood DR (2007). Estimating the sustained swimming ability of coral reef fishes.. Marine and Freshwater Research.

[36] Tittensor DP, Micheli F, Nystrom M, Worm B (2007). Human impacts on the species-area relationship reef fish assemblages.. Ecology Letters.

[37] Arena PT, Jordan LKB, Spieler RE (2007). Fish assemblages on sunken vessels and natural reefs in southeast Florida, USA.. Hydrobiologia.

[38] Ault JS, Smith SG, Bohnsack JA, Luo JG, Harper DE (2006). Building sustainable fisheries in Florida's coral reef ecosystem: Positive signs in the Dry Tortugas.. Bulletin of Marine Science.

[39] Rowat D, Speed CW, Meekan MG, Gore MA, Bradshaw CJA (2009). Population abundance and apparent survival of the Vulnerable whale shark Rhincodon typus in the Seychelles aggregation.. Oryx.

[40] Arzoumanian Z, Holmberg J, Norman B (2005). An astronomical pattern-matching algorithm for computer-aided identification of whale sharks *Rhincodon typus*.. Journal of Applied Ecology.

[41] Bradshaw CJA, Mollet HF, Meekan MG (2007). Inferring population trends for the world's largest fish from mark-recapture estimates of survival.. Journal of Animal Ecology.

[42] Gubili C, Johnson R, Gennari E, Oosthuizen WH, Kotze D (2009). Concordance of genetic and fin photo identification in the great white shark, Carcharodon carcharias, off Mossel Bay, South Africa.. Marine Biology.

[43] Domeier ML, Nasby-Lucas N (2007). Annual re-sightings of photographically identified white sharks (Carcharodon carcharias) at an eastern Pacific aggregation site (Guadalupe Island, Mexico).. Marine Biology.

[44] Buray N, Mourier J, Planes S, Clua E (2009). Underwater photo-identification of sicklefin lemon sharks, Negaprion acutidens, at Moorea (French Polynesia).. Cybium.

[45] Bansemer CS, Bennett MB (2008). Multi-year validation of photographic identification of grey nurse sharks, Carcharias taurus, and applications for non-invasive conservation research.. Marine and Freshwater Research.

[46] Van Tienhoven AM, Den Hartog JE, Reijns RA, Peddemors VM (2007). A computer-aided program for pattern-matching of natural marks on the spotted raggedtooth shark Carcharias taurus.. Journal of Applied Ecology.

